# A long way from Frome: improving connections between patients, local services and communities to reduce emergency admissions

**DOI:** 10.1186/s12875-024-02557-4

**Published:** 2024-08-17

**Authors:** Kathleen Withers, Karen Pardy, Lynne Topham, Rachel Lee, Amir Ghanghro, Hazel Cryer, Huw Williams

**Affiliations:** 1 CEDAR Centre for Healthcare Evaluation, Device Assessment and Research, Cardiff & Vale Ubniversity Health Board, Cardiff Medicentre, Heath Park, Cardiff, CF14 4UJ Wales, UK; 2 Cardiff University, Cardiff, Wales UK; 3 Welsh Value in Health Centre, DDTIV, NHS Executive, Llantrisant, Wales UK; 4 Cardiff South West Primary Care Cluster, Cardiff, Wales UK; 5 ACE (Action in Caerau and Ely), Cardiff, Wales UK

## Abstract

**Background:**

Low socio-economic status can lead to poor patient outcomes, exacerbated by lack of integration between health and social care and there is a demand for developing new models of working.

**Aim:**

To improve connections between patients, local services and their communities to reduce unscheduled admissions.

**Design and setting:**

A primary care cluster with areas of high deprivation, consisting of 11 general practices serving over 74,000 people.

**Method:**

A multi-disciplinary team with representatives from healthcare, local council and the third sector was formed to provide support for people with complex or social needs. A discharge liaison hub contacted patients following hospital discharge offering support, while cluster pharmacists led medicine reviews. Wellbeing Connectors were commissioned to act as a link to local wellbeing and social resources. Advance Care Planning was implemented to support personalised decision making.

**Results:**

Unscheduled admissions in the over 75 age group decreased following the changes, equating to over 800 avoided monthly referrals to assessment units for the cluster. Over 2,500 patients have been reviewed by the MDT since its inception with referrals to social prescribing groups, physiotherapy and mental health teams; these patients are 20% less likely to contact their GP after their case is discussed. An improved sense of wellbeing was reported by 80% of patients supported by wellbeing connectors. Staff feel better able to meet patient needs and reported an increased joy in working.

**Conclusion:**

Improved integration between health, social care and third sector has led to a reduction in admissions, improved patient wellbeing and has improved job satisfaction amongst staff.

**Supplementary Information:**

The online version contains supplementary material available at 10.1186/s12875-024-02557-4.

## Background

It is acknowledged that healthcare services in the UK need to adapt, focusing attention on prevention and improved provision of care closer to home. Benefits of integration throughout the NHS and between health and social care is not a new concept and can lead to better outcomes [1, 2], and numerous exemplars exist [2].

The relationship between social factors and health is well documented [3–5] highlighting the impact of social isolation, loneliness and socio-economic status on healthcare use and patient outcomes. Arguably, traditional mechanism of social care provision has led to variation in services provision, making integration with healthcare more difficult [6]. However, there is growing interest in ‘social prescribing’, linking people to community and voluntary sector support and services to address non-medical needs. Indeed, a recent scoping review identified 159 social prescribing programmes across a number of countries across Europe, North America and Oceania alone [7]. In England, a number of initiatives such as the NHS Long Term Plan [8] and Proactive Care Guidance [9] set out to improve the approach to coordinated multi-professional care. Following a 2018 Parliamentary Review [10] to identify key challenges facing health and social care, the Welsh Government also set out plans to revolutionise care delivery in Wales [11], aiming to bring health and social care together, with a National Transformation Programme to facilitate change. This initiative included targeted funding to support new models of partnership between health, social care and third sector (e.g. non-profit organisations, charities, voluntary and community groups). One project which won early support from this fund was “An Accelerated Cluster Model” led by the Cardiff South West Primary Care Cluster (CSWPCC).

In Wales, primary care clusters include groups of neighbouring general practices which work together to deliver health and care services. There are currently 60 primary care clusters in Wales [12]. These vary in their provision and deliver different services dependent on the local need. While their aim is to bring together services involved in health and care to promote wellbeing of individuals and communities [11], traditionally there has been little or no integration between health, third sector, and social care services.

### Aim

This project aimed to reduce emergency admissions for patients registered with practices in the CSWPCC focusing on developing multi-disciplinary working, and an emphasis on improved connections between patients and communities, and across services. Mechanisms included enhanced delivery and provision of social prescribing initiatives, a focus on prevention by identifying people who are at risk and actively supporting them to remain as independent as possible (e.g. by reducing unscheduled admissions and readmissions), improved interagency working (i.e. between health, social care, and the third sector), and improved advance care planning.

The scope of the new service provision was based on the Frome Compassionate Communities model [13]. Its goals were:

- Implement asset-based community development at cluster level.
- Developing workforce well-being.
- Identifying at risk individuals and actively supporting them to remain as independent as possible.
- Ensure personal needs are prioritised through individual care plans.
- Establish a multidisciplinary team (MDT) to support vulnerable individuals.
- Establish a cluster discharge liaison hub.
- Develop a cluster model to maximise opportunities for seamless working and allocating resource based on population needs.

These focus on the implementation of four key elements: a strong community MDT meeting regularly; an integrated care hub supporting patients on discharge from hospital; community development and social prescribing; and advance care planning.

This paper aims to describe the implementation and roll out of the programme, and provide feedback on the available outcomes.

### Design and setting

The CSWPCC was established in 2014, with a core membership of 11 GP Practices serving an ethnically diverse population of approximately 74,000 people. There are high levels of deprivation within the cluster and over 45% of the population live in the 20% most deprived areas of Wales [14]. Additionally, 2016–2018 data suggests people living in the South West Cardiff cluster area are more likely to smoke and less likely to be a healthy weight than the general population in Wales [15]. One of nine GP Clusters within Cardiff & Vale University Health Board (CAVUHB) area (which provides healthcare services to over 470,000 people in the Cardiff and the Vale of Glamorgan), it had one of the highest hospital admission rates within the area at 32% above the average.

The CSWPCC were keen to implement lessons from previous schemes [16], particularly from the Frome “Compassionate Communities” project [13]. The Frome model focused on person centred care, social prescribing, development of community assets, and enhanced patient review following hospital discharge, and led to a reduction in hospital admissions of 14% [13].

#### Funding

Transformation Fund grant funding of £1,287,463 was awarded to this project early in 2019.

### Methods

#### Multidisciplinary team (MDT) setup

Historically, within the South West Cluster, organisations were working in silos with little or no interaction. In February 2019, leads within the cluster implemented biweekly MDT meetings, aiming to identify support for patients with complex or social needs, bringing together GPs, cluster pharmacists and staff from agencies including the local council, CAVUHB, community services, mental health teams and third sector groups. With documented patient consent, GP’s would bring cases to meetings for discussion and an opportunity for teams to offer support. There were no strict referral criteria, or limit on the number of available slots, and any MDT member was able to refer patients. The purpose of the MDTs was to bring different individuals and groups with a wide range of skills together, allowing the cluster to provide patients with medical and non-medical solutions to their problems. Integrated IT systems were made accessible to partner groups allowing information to be shared between key stakeholders with formal information governance arrangements, and standard operating procedures around this. Over time the attendance at these meetings expanded with representatives from groups detailed in Table 1.

**Table 1:** MDT attendee groups

Group	Descriptor
ACE (Action in Caerau and Ely)	A community charity located in the West of Cardiff, ACE works with local communities to deliver a range of services and activities
Cluster pharmacists	Works with a group of practices to achieve primary care cluster priorities. Also provides cluster support in relation to prescribing trends and analysis depending on cluster priorities
Care & Repair	A Welsh charity, working to ensure that older people can live independently in safe, warm, and accessible homes
CRT (Community Resource Team)	An integrated Health, Social Care and Third Sector Team working with people in their own home to maximise functional independence in activities of daily living. This team included an occupational therapist, physiotherapist and nurse.
General Practitioners	GP representatives from within the cluster
Independent Living Services	A Cardiff Council initiative providing advice or assistance on living independently. Advice on benefits, home adaptations and equipment social care, safety at home and more is available.
Mental Health for Old People	Representatives from older peoples mental health services
Mental Health team	Representatives from mental health services
Palliative care services	Made up of different healthcare professionals to co-ordinate the care of people with an incurable illness. As specialists, they also advise other professionals on palliative care.
Wellbeing4U	Wellbeing 4U uses a Social Prescribing model to deliver public health priorities through social intervention. It is able to offer a mixture of outreach, one-to-one work and signposting to community activities and the third sector
District nurses	Registered nurses who work within the community, e.g. in people’s homes, in primary care settings and care homes

As well as providing advice and support on complex health issues, groups provide practical support with home adaptations, improvements and repairs, meal provision, medication reviews and prescription collection. They also give advice on housing, benefits and debts, managing energy and food costs, grant opportunities, substance misuse, wellbeing, and opportunities to connect with community and social groups. Transformation funding allowed GP time to be backfilled allowing them to attend the MDT’s. Other roles were already funded but time was prioritised for staff to attend. Meetings moved online in March 2020 in response to the COVID-19 pandemic.

#### Discharge liaison/well-being hub

A discharge liaison hub was established in September 2019, with dedicated staff equivalent to two full-time administrators and a co-ordinator financed by the transformation fund. The Hub also hosted a dedicated worker from Independent Living Services (ILS), and occupational therapist and pharmacists, enhancing access. The initial aim of the Hub was to identify and contact potentially vulnerable patients following hospital discharge. These individuals were telephoned within 48 h of discharge allowing them time to settle back home and identify unmet needs. Hub staff were then able to offer appropriate support from the stakeholders attending the MDT meetings. This gave patients access to support to live independently, e.g. by installing ramps and hand rails, or accessing ‘meals on wheels’. Cluster pharmacists provided standardised medicines reconciliation of patient discharge summaries allowing medication issues to be identified and resolved promptly. This marked a significant change from the standard of care where this work was carried out ad hoc by practices, usually by the duty GP actioning medication changes, and no wellbeing call to the patient after discharge.

COVID-19 saw the focus of the Hub extend to provide emotional support and linking patients with community groups offering services including prescription collection and food shopping.

#### Community wellbeing connectors

Wellbeing Connectors were newly commissioned from existing social prescribing partners, to improve capacity and develop capability to self-care. Unlike similar models, team members were employed by community organisations, not directly by CAVUHB. Staff were able to identify and fill gaps in wellbeing resources, activities and services in the local area. They have been able to work across the cluster supporting patients to develop and maintain community links and improve their own and one another’s lives. As well as being a contact for isolated and vulnerable patients, connectors offer people a range of services to improve well-being including bereavement peer support, coffee mornings, women’s only exercise classes, men’s group, and gardening club. Some offer support to particular groups such as those who speak English as a second language. Patients were also supported with digital inclusion to help them feel more connected.

#### Advance care planning (ACP)

Living with a chronic or life limiting illness can lead to uncertainty, and there are challenges in ensuring patients’ wishes are accommodated when these are not well defined. Having open, honest conversations about what is important to an individual and what they want in the future and documenting this clearly, can help make sure these wishes are met. To support ACP within the cluster, extensive training was undertaken, including practice and care home staff, and recording processes were formalised via a template embedded within the clinical system. Information on ACP was included in the Cluster newsletter which is available to all patients and engagement events were held across the cluster including in two local Mosques. A Macmillan community development worker was separately funded at this time and supported this area of work, being regularly available in practice waiting rooms and providing direct support for those patients on palliative care registers. This provided interconnections within existing services, including hospice at home, and linking in with the cluster practices to reduce silo working and improve communications. ACPs allow patients to record their wishes on future care including preferred place of care, and place of death and ensures they are shared with loved ones and healthcare professionals.

### Data collection

Key outcomes were collected using mixed methods. Quantitative data was collected on the number of patients contacted by staff at the Hub, and the wellbeing team, the number discussed at MDT meetings, and the number of GP visits pre- and post-discussion at MDT. Other data collected included: number of medicines reconciliations, referrals to mental health services and other organisations, and the number of signposting suggestions. The rate of GP referrals to secondary care assessment units was available throughout the project with statistical process control charts to monitor trends as per standard Quality Improvement methods [17]. Assessment units are the first point of entry for patients referred to hospital as an acute medical/surgical emergency by their GP. Hospital bed days was collected continuously throughout the time period by a third party provider, Lightfoot Solutions UK Ltd., separately commissioned by CAVUHB.

Feedback was also collected from service users accessing the MDT, and those interacting with the Wellbeing connectors via the Short Warwick-Edinburgh Mental Wellbeing Scale (SWEMWBS), which measures mental wellbeing in the general population. In-depth qualitative interviews conducted by an independent researcher (KW) were undertaken with 27 staff, including representatives from the cluster, the council and third sector. These involved GP’s, pharmacists, occupational therapists, project support and admin staff, and operational managers. Participants provided informed consent including consent to publication of findings. Topic guides were developed to guide these interviews and are available in the supplementary material. Data were analysed thematically analysed using NVivo.

## Results

**Fig. 1:**
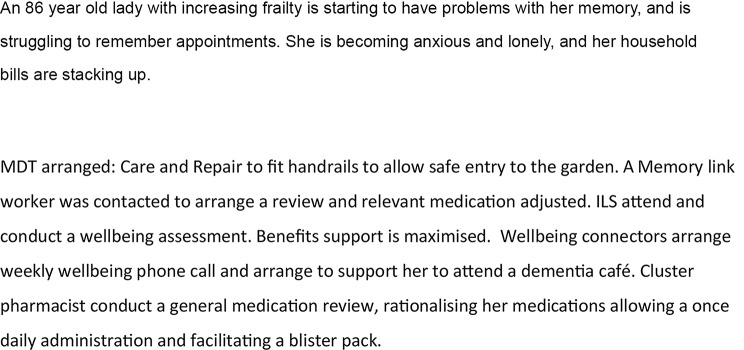
Case example

In the years 2019–2020 and 2020–2021, against a rapidly changing background due to the COVID-19 pandemic, MDT meetings discussed 354 and 238 unique patients respectively (Table 2). Detailed demographic data are not available. However, generally those discussed were older people with frailty and struggling to manage at home, or people with housing, social, isolation and mental health needs. A common example of support provided is available in Fig. 1.

**Table 2:** Patients reviewed by the MDT, and services they were referred to by group

	2019				2020				2021				2022				2023	Totals
	2019	2019/2020				2020–2021				2021/2022				2022/2023				
	Feb-Mar	April-June	July-Sept	Oct-Dec	Jan-March	April-June	July-Sept	Oct-Dec	Jan-March	April-June	July-Sept	Oct-Dec	Jan-March	April-June	July-Sept	Oct-Dec	Jan-March	
Total Patients Reviewed	95	160	211	240	225	97	128	143	82	125	168	150	193	142	126	102	145	2532
New Patients	59	73	86	104	91	49	75	72	42	59	81	78	95	77	74	65	103	1283
Follow up Patients (previous MDT review)	36	86	125	135	134	48	47	71	39	66	87	72	98	65	52	37	42	1240
New Patients needing further follow up	56	62	71	89	74	19	37	38	21	25	31	40	38	34	20	12	16	683
Patients Discharged	29	70	78	104	98	52	56	75	45	51	89	69	98	79	82	67	89	1231
**Services Patients Referred into**																		
Caring Needs / Housing	35	52	43	52	51	20	21	21	13	14	24	10	25	19	31	12	19	462
Physio / OT	20	27	18	19	18	7	19	10	10	24	31	39	43	17	15	9	22	348
Social prescribing / Befriending	13	19	26	30	20	12	15	34	7	28	23	33	48	25	22	8	22	385
Mental Health	0	6	3	8	9	13	5	2	8	7	4	16	20	9	11	7	11	139
Pharmacist	6	19	14	24	9	3	2	7	2	8	6	8	7	3	7	3	5	133
Other*	7	7	4	2	2	0	0	0	0	0	2	0	0	0	0	2	0	26

* Includes palliative care services, safeguarding, district nurse, dietician and Velindre Cancer Centre

GP records show that patients referred to MDT meetings contact their surgery 20% less after their case has been discussed. The MDTs also helped streamline referral processes. During the 2020–2021 period, in the midst of the pandemic, Hub staff contacted 4,740 patients. In the same year, cluster pharmacists completed 3,050 medicine reconciliations, with reduction in variation through standardised read coding of outcomes from discharge summaries. Approximately 1 in 20 discharge summaries needed clarification with medication issues including insufficient medications issued on discharge, changes to medications with insufficient information, and lack of arrangements for monitoring.

Wellbeing Connectors supported over 200 patients April-Sept 2020 with some outcomes detailed in Table 3. WEMWEBs feedback during this time found 85% of responders reported an improved sense of wellbeing. A number stated the contact was very beneficial with one commenting: ‘*It’s been invaluable, it’s been a lifeblood. Knowing that you are going to call has kept me going…. Without those things I would feel much more alone’*. While many of these services were specific to the COVID-19 pandemic, general support has focused on identifying what is important to a person and helping them to achieve that. Examples include signposting and providing support for people to attend activities (e.g. transport and chaperoning) setting up a neurodivergent support group, and running a women’s exercise group.

**Table 3:** Wellbeing connector support provided

No of patients assisted	Type of assistance
16	Food sourced while shielding or self-isolating via food pantries and shopping services
78	Prescriptions collected and delivered (many of these receiving multiple deliveries)
43	Provided with items to support well-being such as craft kits and activities
64	Signposted to other support services
51	Help provided to resolve issues with debt, benefits, and housing support
11	Supported with digital inclusion through the provision of devices, internet access and advice

In 2020–2021, ACP discussions had started, with 139 decisions recorded, 15 of these noting preferred place of death.

### Staff feedback

Staff feedback from all agencies involved was overwhelmingly positive, with qualitative findings indicating that the new collaborations are offering patients a wide range of support in a systematic and holistic way. Interviewees suggested that improved working relationships meant many patients had benefitted. Cluster staff reported being more aware of resources and services available, meaning they were better equipped to resolve issues for those not referred to the MDT. Some GPs felt their general approach had changed as they were able to manage their patients’ social issues in a way that had not previously been possible. There was improved joy in working across the teams involved, with GPs in particular reporting improved job satisfaction. Illustrative quotes are available in Table 4.

**Table 4:** Illustrative quotes

Collaborative working	Cluster GP 1	‘A good MDT meeting just ….is blinding…. I would not have made 10 referrals at once but this is definitely going to get them the care they need as quickly as possible’
Improved knowledge	Cluster GP7	‘Once you know what is there you don’t need to take everybody to the MDT, you can refer them directly’
Joy in working	Cluster GP2	‘… this is the most excited I’ve been in a long time about my job because it’s actually… it’s what I thought general practice would be about when I started, which was looking at people holistically.…. this has totally rejuvenated my experience of general practice’
Improved service provision & faster resolution	Cluster GP1	‘I’ve got total confidence that they are definitely doing a better job than I would have done by seeing them 5 or 10 times over a drawn-out period. You just know that it’s going to be sorted and there’s going to be a really good outcome for the patient which is great. And as the GP who has seen them and then “Oh I’ll involve my MDT” you feel like you come across as this superpower … “Oh I’ll get that sorted for you, don’t worry!”’

### Reduced referrals and admissions

GP referrals to assessment units had risen steadily throughout CAVUHB over the two-years prior to the funded interventions, to an average of 56 per 10,000 of the population in Jan 2018-Jan 2019. At this time CSWPCC was above this at 63 per 10,000. Following the changes, this fell to 60 per 10,000 between Feb 2019-Feb 2020 (a change of 3%), against a background rise of 3% to an average of 59 per 10,000. This improvement has been sustained with the CSWPCC referrals in June 2023 remaining below the CAVUHB average.

**Fig. 2:**
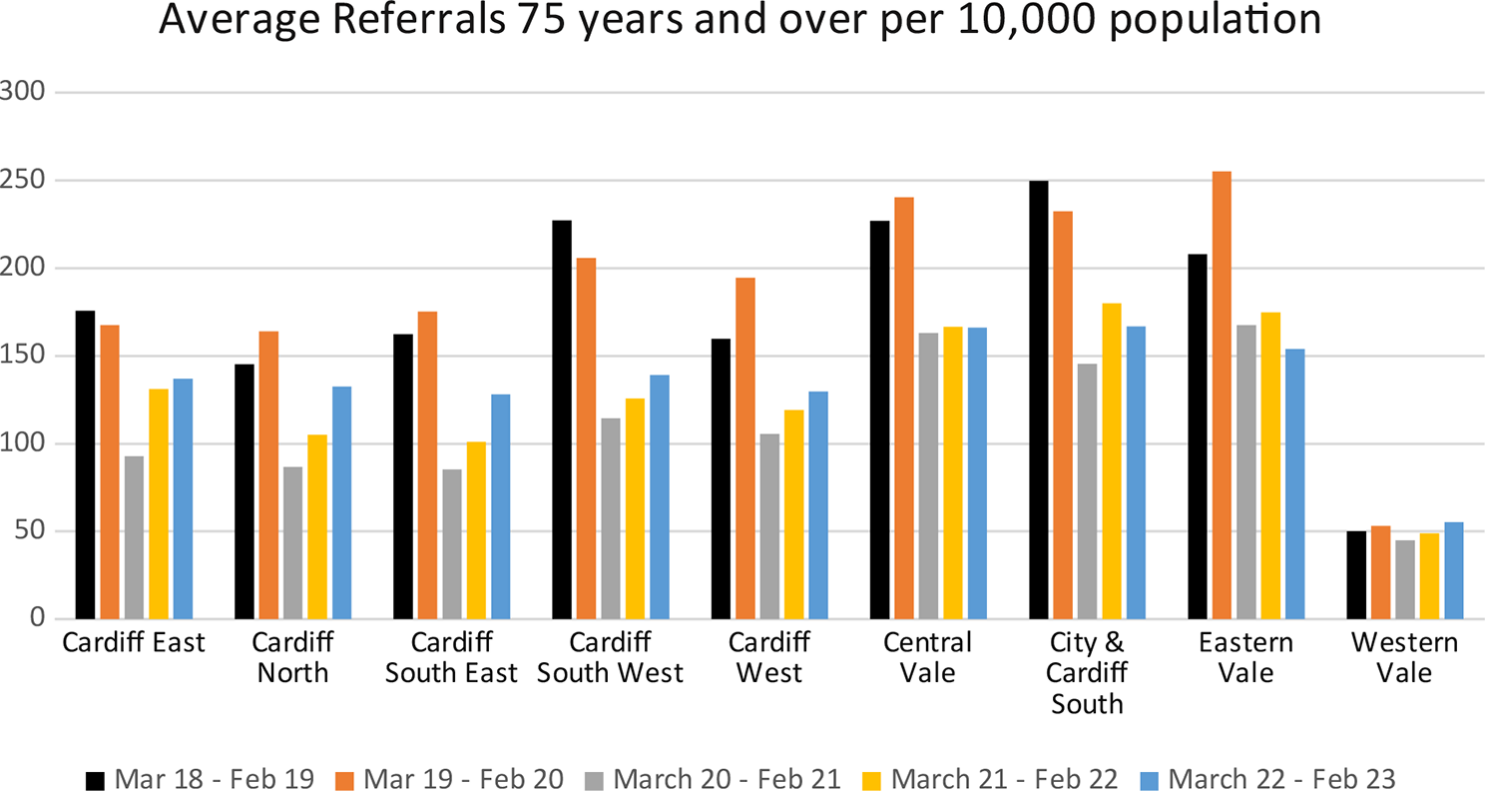
GP Referrals to assessment unit for people aged over 75

Improvements in unscheduled admissions for patients aged 75 and over was particularly notable. Prior to the intervention, in the year March 2018-February 2019, the average CAVUHB GP referral to assessment units per 10,000 of population aged 75 and over was 178/month. The average for the South West Cluster was the joint second highest in the area at 227 (27% above average). In the year following the intervention, average GP referrals rates for this group continued to rise across CAVUHB. Three clusters were the exception; South West reduced their rate by 9% to 206 referrals per 10,000 population, while City & Cardiff South and Cardiff East reduced by 7% and 5% (250 per 10,000 to 232 per 10,000, and 176 per 10,000 to 168 per 10,000) respectively. This improvement continued post-COVID with a further drop. While other clusters showed a decrease during this time, the decrease for the South West cluster was largest, with a reduction of 50% from baseline in the year March 2020-Feb 2021. This improvement against baseline has been maintained, with 2022–2023 data showing that the South West Cluster has 39% lower rate of referrals per 10,000 compared to pre-intervention levels (139 compared to 227 per 10,000) (see Table 5). With the current cluster population of 74,000 this change equates to over 800 avoided monthly referrals to assessment units for the cluster as a whole.

**Table 5:** Change in average referals from March 2018-Feb 2019 by cluster

	March 2019 - Feb 2020	March 2020 - Feb 2021	March 2021 - Feb 2022	March 2022 - Feb 2023
Cardiff East	−5	−47%	−25%	−22%
Cardiff North	13%	−40%	−28%	−9%
Cardiff South East	8%	−47%	−38%	−21%
Cardiff South West	−9	−50%	−45%	−39%
Cardiff West	22%	−34%	−26%	−19%
Central Vale	6%	−28%	−27%	−27%
City & Cardiff South	−7	−42%	−28	−33%
Eastern Vale	23%	−20%	−16%	−26%
Western Vale	6%	−10%	−2%	11%

Monthly emergency bed days, per head of population also decreased following changes implemented. In 2018, the CSWPCC population as a whole had over 6,200 emergency beds days/month (equivalent to attendances), the second highest among CAVUHB Clusters. From January 2019-February 2020 (post-implementation but before the impact of COVID-19), there was a decrease of 17% similar to other clusters, however post-COVID this decreased more significantly than other clusters dropping further to 3,099 bed days/month for the South West Cluster, a total decrease of 50%, bringing it to below CAVUHB average. South West Cluster bed days reduced by an average of 538/month from February 2019 to January 2020 compared to the counterfactual trend. Based on £595/bed day cost this is the equivalent of saving of over £3.8 million/annum for this cluster [18].

### Barriers and facilitators

Interviews with staff involved in setting up the different elements of the programme, identified a range of enablers, barriers and learning opportunities. Previous examples of service transformation projects such as the Frome model (Abel, 2018), provided inspiration, and dedicated funding gave staff protected time, and allowed posts including a project manager and hub facilitators to be created. On a practical basis, having an independent chair and note-taker was helpful in facilitating MDTs. High-level support from executive teams across key organisations was a key enabler overall. Indeed, general ‘buy-in’ from staff was noted as essential to getting the programme of work started, with staff across all sectors engaging with the MDTs and Hub as the first changes were implemented. This allowed things to develop in an intuitive way as the group identified what was needed to progress planned changes in the most effective way. ‘*I think it was the openness and the willingness to try something new that really helped with that*,* but having the protected time to do it was vital*’.

The enthusiasm of several GPs and partners in other organisations linked to the cluster drove the project forward, with their willingness to listen to others seen as useful in adapting the programme successfully through its duration. For example, in Frome each GP practice had dedicated lead, while in this model, the work was facilitated on a cluster basis by the hub coordinator. Initially this role was fulfilled by a member of the ILS team, however it was recognised that this resource was best utilised elsewhere, and the role is now fulfilled by a care navigator. Additional iterative changes were made, for example, initially patients were contacted prior to discharge from hospital to enquire whether they needed additional support. However, Wellbeing coordinators found that this offer was best made several days after discharge, once patients had been able to settle back home and identify their new needs. MDT meetings also moved from face to face to online to facilitate attendance.

Despite general support for the work, several staff groups without protected time found it more difficult to attend MDT meetings and time pressures of an additional meeting were a challenge. Having meetings on consistent days was helpful for scheduling, but challenging for those with regular diary clashes, particularly part-time staff. The move to online MDTs removed travel time and allowed staff to briefly dip into meetings. Allowing staff to present a case on behalf of colleagues also helped.

Effective IT systems which allowed individuals from some different teams to access patient notes helped with information sharing and allowed staff to see what individual patients’ challenges were and what options had been considered. A dedicated platform was procured for facilitating social prescribing referrals enabling support directly from the patient clinical record. However, Information Governance (IG) around this was seen as the biggest hurdle in setting up and maintaining the changes implemented, particularly related to teams external to the NHS. In some cases this prevented staff working efficiently and cohesively. It was more time consuming than anticipated to identify and meet the varied IG requirements. Having dedicated capacity from someone with appropriate expertise in this area would have been beneficial as it took a great deal of time to understand what was needed from and for all of the organisations involved.

## Discussion

Over the course of the funded programme of implementation, all project aims were met with the development of the cluster model, discharge liaison hub, and MDT facilitating the clusters ability to identify and support at risk individuals. The personal needs of the most vulnerable patients are better supported by improved individual care planning and interdisciplinary working. This holistic approach has led to improved staff morale.

Although a formal evaluation of the programme has not been undertaken, outcome data is available for some aspects of changes implemented. The background of COVID-19 led to CAVUHB implementing other changes at speed from early 2020, making direct comparisons difficult. However, the CSWPCC Transformation Programme has led to a reduction in emergency admissions and bed days with significant related cost avoidance. This reduction is likely to be due to a combination of factors including proactive support provided to patients deemed vulnerable to deterioration via the MDT which utilised a wider range of services than traditionally available to GP practices, including social prescribing networks, social care services, and community reablement teams. Initiatives provided assistance to people isolated or lacking in support, working with existing networks, and enhancing them when gaps were identified. Following hospital discharge, the team Hub focused on enhancing support systems once patients had settled back home. The team of pharmacists ensured timely and accurate medicines reconciliation processes, mitigating the documented risk inherent in the transfers of care during discharge [19], identifying errors and potential harms in a significant proportion of the summaries received. A focus on advance care planning, especially in care-home settings, with training and awareness raising led to a clearer communication of patients’ wishes at the end-of-life which may contribute to a reduction in unnecessary conveyances to hospital settings.

Inviting groups to work with healthcare services was met with almost universal, enthusiastic acceptance. Many organisations had been trying to engage with primary care or the wider health system for some time. Historically, organisations were working in silos with little or no interaction. The Hub work was done in different ways ad hoc by practices usually by the duty GP on that day actioning medication changes and no wellbeing call to the patient after discharge. This project has provided a solid framework to facilitate collaborative working, resulting in an improved offer to patients. The model is now embedded as business-as-usual in CSWPCC and is now being introduced to all other clusters across CAVUHB.

The CSWPCC transformation work was of particular benefit during the COVID-19 pandemic as collaborative working and support systems were already in place. This meant that teams were well placed to facilitate social and wellbeing support for those that were most vulnerable and shielding.

## Strengths and limitations

The large population and involvement of numerous GP practices strengthens the reliability and generalisability of this programme of work and suggests that lessons learned from the Frome project are scalable. As no practices or patients were excluded, the risk of bias is reduced, however, as no comparator group is available, causation is difficult to prove. The programme would also benefit from a full mixed methods evaluation. The impact of COVID-19 led to significant changes in the delivery of healthcare nationwide, and also led to a shift service use. It has therefore not been possible to provide a simple ‘before and after’ analysis to measure the impact of the interventions. Additionally, a number of other initiatives were implemented at different clusters and more widely during the timeframe, and these may also have impacted on some of the outcomes.

### Comparison with existing literature

Similar programmes of work both within the UK and further afield report varied but comparable results when implementing social prescribing initiatives. These include improved care and social networks, increased wellbeing and a reduction in unplanned hospital admissions [13, 20–23]. While the methods of implementing social prescribing are varied, they are most often delivered through healthcare, while including a range of non-medical collaborators [7] as in the South West Cluster model.

### Generalisability and shared learning

This method of integrated health and social care to provide preventative support to a large population has proven to improve staff and patient wellbeing and reduce GP attendance and hospital admissions. Many of the lessons learned are generalisable to other projects, particularly collaborative projects between health and social care. A number of key factors were identified in the qualitative feedback, and these were critical to the success of this project, and should be considered for those undertaking similar programmes of work.

As noted, executive leadership support across the involved organisations was key. Senior leaders in CAVUHB, Cardiff Council, CSWPCC (and individual practices), and third sector partners were on-board and facilitated engagement in various aspects of the work. Similarly, funding was important, both for new roles and existing individuals time to support implementation. Without this, due to the intensity of public sector working and the challenges of delivering complex change, the project would not have happened. Another essential resource included the space to carry out the work.

Committed funding over a 2-year period with an understanding that if the project showed gains the funding would continue, meant the project team could fully commit to delivering the objectives. The outcomes delivered show the value of dedicated funding for developing and delivering new models of care.

Using aspects of an existing model, but adapting it to local needs can provide a basis for learning while retaining flexibility. For example, initially the work of the discharge hub was envisaged to be carried out in each practice in the cluster. It soon became apparent that doing it on each site was inefficient and there was a lack of capacity to support this method, therefore a central hub was developed. Additionally, building on existing contacts where available reduced the need to identify new partners and develop new relationships.

The project team itself was a major driver to the success of the programme, and identifying and including engaged multidisciplinary members was key. Initial weekly meetings allowed the program methodology to develop and build relationships, but these decreased to fortnightly, then monthly once processes were established. A potential issue for membership of the team was the ability to recruit quickly into posts. As different organisations were involved, the HR practices varied and this limited recruitment and employment flexibility, leading to a lack of agility at times. In addition, fixed-term contracts proved challenging to recruit into and a succession of short-term posts worked against developing good working relationships.

Communications with the wider team was essential, and regular engagement and feedback with involved practices and organisations is needed. Whilst all practices in the cluster have engaged and now referrer to MDT meetings, it took time to engage some groups/individuals. Regular sharing of successes, pen-pictures and case studies helped drive engagement, and slots at educational events helped to promote the model. Early and widespread communications are recommended for other multi-party projects.

Information governance was a significant challenge, and should be considered at the earliest stage. A lack of clear information sharing agreements initially led to situations where MDT members were unable to share useful clinical information with colleagues, and occasionally third sector partners needed to be excluded from discussions. IG issues also affected the data collection plan, preventing data linkage which would have supported the evaluation. Data capture itself was a challenge, although the involvement of Lightfoot as a dedicated resource to collect and analyse data was helpful. While the issues around data and IG were resolved over time, an early focus on these, ideally with support from experienced colleagues would have saved time and improved processes. National level agreements for IG would facilitate the roll out of similar models.

This was a complex and ambitious project, particularly as it was delivered throughout a global pandemic. It has illustrated numerous opportunities for shared learning, and has been an exemplar of integrated working. The cluster are able to identify and support vulnerable patients more effectively, and work with stakeholders to provide a range of medical and non-medical solutions to their population. Community connectors have supported a large number of patients, reducing isolation, making them feel more connected which was particularly important during the pandemic. The project also impacted positively on the cluster workforce as their perceived ability to support their patients has grown. The cluster continue to deliver the services as ‘business as usual’, whilst continuing to develop cross-sector working to support patient care.

### Electronic supplementary material

Below is the link to the electronic supplementary material.

Supplementary Material 1

## Data Availability

The datasets used and analysed during the current study are available from the corresponding author on reasonable request.
