# Supplementary material for: A long way from Frome: improving connections between patients, local services and communities to reduce emergency admissions
Source: BMC Prim Care. 2024 Aug 17;25:307. doi: 10.1186/s12875-024-02557-4 (PMC11330060; doi:10.1186/s12875-024-02557-4)
Supplement: Supplementary file 1 — Supplementary Material 1 [file 12875_2024_2557_MOESM1_ESM.docx]

**Staff Topic Guide - Community based MDT**

Thank you for taking the time to speak to me today. As you know, my name is Kathleen, I am a research scientist who works for the NHS, based at Cardiff & Vale. Although I am supporting the Cardiff South West Cluster transformation project, I work independently to the team.

We are trying to find out what staff and patients think about the changes that have been made in relation to the MDT, and how it affects care, and your role. We are really interested to hear what you think of it and want to know the good and the bad so please be as honest as possible.

**Anything we discuss will be anonymous, but we may use anonymised quotes in reports and feedback we provide** I will be taking notes during our conversation, but so that I don’t miss anything important it would be helpful for me to record our conversation. Would you be happy for me to record our conversation?

Yes / No

1. From your viewpoint what were the enablers for setting up the MDT?
2. What went well in setting it up?
3. What were the barriers? (Information Governance, managerial buy-in, resource)
4. What infrastructure was needed to get it to work? Physical Space. IT (Vision 360, PARIS access, Social Services IT system and more) Appointment system
5. What additional infrastructure would make it work better?
6. Who are the key stakeholders?
7. What happens in a “good” MDT meeting? Full attendance/Strong Chair/glitch free IT/referrals made in good time?
8. What happens when it goes less well? Internet issues/low attendance/weak chair/focus on extraneous issues?
9. Anything else to add?

**Staff Topic Guide - Community Wellbeing/Discharge Hub**

Thank you for taking the time to speak to me today. As you know, my name is Kathleen, I am a research scientist who works for the NHS, based at Cardiff & Vale. Although I am supporting the Cardiff South West Cluster transformation project, I work independently to the team.

We are trying to find out what staff and patients think about the changes that have been made in relation to the Community Wellbeing / Discharge Hub, and how it affects care, and your role. We are really interested to hear what you think of it and want to know the good and the bad so please be as honest as possible.

**Anything we discuss will be anonymous, but we may use anonymised quotes in reports and feedback we provide** I will be taking notes during our conversation, but so that I don’t miss anything important it would be helpful for me to record our conversation. Would you be happy for me to record our conversation?

Yes / No

1. Thinking about the community wellbeing/discharge hub, in your view what has worked well?
2. Who do you think are the key members of the Hub?
3. What have been the enablers?
4. What have been the barriers?
5. What benefits have there been for patients?
6. What gap in care has the Hub filled?
7. What benefits have there been for GPs/other healthcare professionals?
8. Have there been downsides to the Hub for patients and staff?
9. Any other comments?
